# PMI: A ΔΨ_m_ Independent Pharmacological Regulator of Mitophagy

**DOI:** 10.1016/j.chembiol.2014.09.019

**Published:** 2014-11-20

**Authors:** Daniel A. East, Francesca Fagiani, James Crosby, Nikolaos D. Georgakopoulos, Hélène Bertrand, Marjolein Schaap, Adrian Fowkes, Geoff Wells, Michelangelo Campanella

**Affiliations:** 1 Department of Comparative Biomedical Sciences, The Royal Veterinary College, University of London, Royal College Street, London NW1 0TU, UK; 2 UCL Consortium for Mitochondrial Research (CfMR), London WC1E 6BT, UK; 3 UCL School of Pharmacy, 29-39 Brunswick Square, London WC1N 1AX, UK

## Abstract

Mitophagy is central to mitochondrial and cellular homeostasis and operates via the PINK1/Parkin pathway targeting mitochondria devoid of membrane potential (ΔΨ_m_) to autophagosomes. Although mitophagy is recognized as a fundamental cellular process, selective pharmacologic modulators of mitophagy are almost nonexistent. We developed a compound that increases the expression and signaling of the autophagic adaptor molecule P62/SQSTM1 and forces mitochondria into autophagy. The compound, P62-mediated mitophagy inducer (PMI), activates mitophagy without recruiting Parkin or collapsing ΔΨ_m_ and retains activity in cells devoid of a fully functional PINK1/Parkin pathway. PMI drives mitochondria to a process of quality control without compromising the bio-energetic competence of the whole network while exposing just those organelles to be recycled. Thus, PMI circumvents the toxicity and some of the nonspecific effects associated with the abrupt dissipation of ΔΨ_m_ by ionophores routinely used to induce mitophagy and represents a prototype pharmacological tool to investigate the molecular mechanisms of mitophagy.

## Highlights

• PMI stabilizes Nrf2 and upregulates the dependent gene product P62
• The increased P62 level drives mitochondrial autophagy
• PMI acts downstream of the PINK1/Parkin pathway leaving the ΔΨ_m_ intact

## Graphical Abstract

**Figure:**
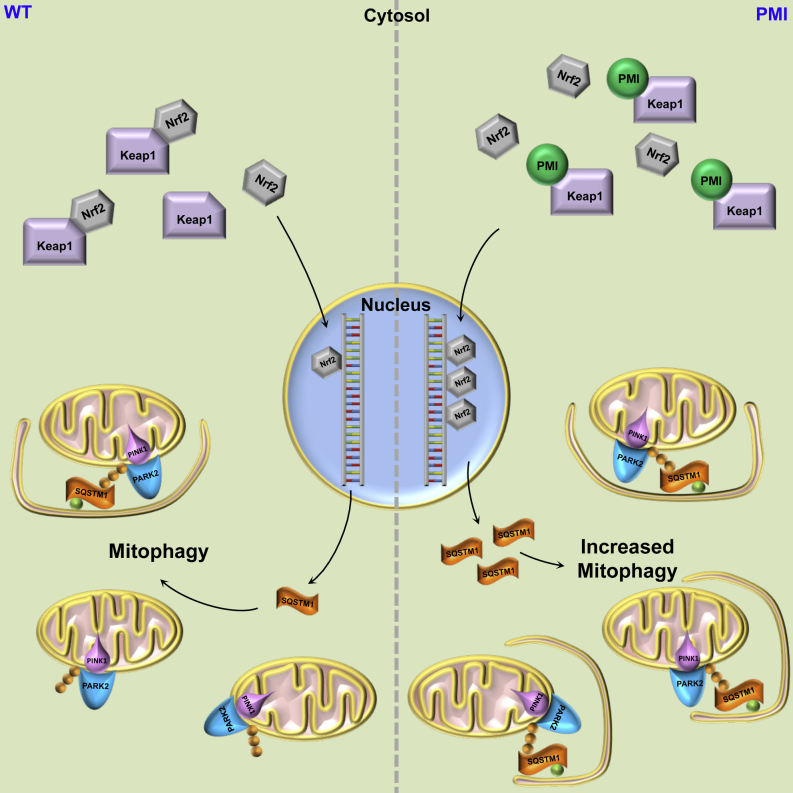

## Teaser

East et al. developed a pharmacological tool, PMI, to investigate the molecular mechanisms of mitophagy. PMI circumvents the toxicity and some nonspecific effects associated with the abrupt dissipation of mitochondrial membrane potential by ionophores that are routinely used to induce mitophagy.

## Introduction

Mitophagy (Kim et al., 2007) is the process by which damaged or dysfunctional mitochondria are selectively engulfed by autophagosomes and delivered to lysosomes to be degraded and recycled by the cell. The most well-recognized mechanism governing the recruitment of autophagosomes to mitochondria is that driven by the PINK1/Parkin pathway (Narendra and Youle, 2011). The PTEN-induced putative kinase 1 (PINK1) accumulates on the outer membrane of dysfunctional mitochondria where it triggers the recruitment of the E3 ubiquitin ligase Parkin (Jin et al., 2010, Narendra et al., 2010, Valente et al., 2004). Once localized to mitochondria, Parkin ubiquitinates several OMM proteins that are consequently targeted by P62/SQSTM1 (Geisler et al., 2010). P62 recognizes ubiquitinated substrates and acts as an adaptor molecule through direct interaction with autophagosome-associated LC3 driving the recruitment of autophagosomal membranes to the mitochondria (Pankiv et al., 2007). Several alternative Parkin-independent mechanisms are also suggested to play a part in mitophagy. Damaged mitochondria can increase FUNDC1 and Nix expression, which may in turn recruit autophagosomes to mitochondria by direct interaction with LC3 (Liu et al., 2012, Novak et al., 2010). Upon mitochondrial depolarization, the ubiquitin ligase Smurf1 also targets mitochondria to induce mitophagy and, recently, the roles of other ubiquitin ligases in mitophagy have been described (Ding and Yin, 2012, Fu et al., 2013, Lokireddy et al., 2012). Currently, there is a lack of practical and specific pharmacological tools to manipulate mitophagy and facilitate dissection of the molecular steps involved in the removal of mitochondria from the network via this pathway. Mitophagy is now recognized as a fundamental process in cellular homeostasis because its deficiency is linked to several neurodegenerative diseases and cancers (de Castro et al., 2010, Karbowski and Neutzner, 2012, Soengas, 2012, Wallace, 2012).

The regulation of P62 expression is partly controlled by the transcription factor Nrf2 (nuclear factor erythroid 2-related factor 2), due to the presence of an antioxidant response element (ARE) in its promoter region (Ishii et al., 2000, Jain et al., 2010). Thus, compounds that induce Nrf2 activity have the potential to enhance P62 expression. A number of electrophilic natural products, including the isothiocyanate compound, sulforaphane, upregulate Nrf2 by interfering with its regulator protein, the redox sensitive ubiquitination facilitator Keap1 (Kelch-like ECH-associated protein 1) (Cheng et al., 2011, Hayes et al., 2010, Kensler et al., 2007). Sulforaphane and related compound 1 covalently modify cysteine residues in the intervening region of Keap1, which disrupts the ubiquitination, with subsequent destruction of Nrf2. This results in increased concentrations of Nrf2 and in the expression of a range of ARE-dependent gene products involved in phase II metabolism (e.g., glutathione synthesis and conjugation enzymes, NQO1, heme oxygenase-1, etc.) (Hayes et al., 2010, Hong et al., 2010, Zhu et al., 2008) and redox control (e.g., thioredoxin, thioredoxin reductase), in addition to P62 (Jain et al., 2010, Lau et al., 2010, Stępkowski and Kruszewski, 2011). Sulforaphane shows activity in a number of preclinical models of disease prevention, including protection against exposure to oxidizing agents and carcinogens (Kensler et al., 2013). However, the isothiocyanate class of compounds, along with other reactive Nrf2 inducing agents, is capable of interacting with a range of other cysteine-containing proteins within the cell, which can make dissecting their biological activity rather difficult. Based on this, we postulated that pharmacological inducers of Nrf2 that lack a covalent binding motif may upregulate Nrf2-dependent gene expression (including *p62*), and thereby have an effect on mitophagy, but may have less potential for cytotoxicity than sulforaphane (cytotoxic at concentrations above 10 μM in mouse embryonic fibroblast [MEF] cells). With this aim in mind, we identified P62-mediated mitophagy inducer (PMI) (HB229) as a molecule that upregulates P62 via stabilization of Nrf2 and promotes mitophagy. The compound appears to bypass the upstream steps of the mitophagic cascade, acts independently from the collapse of the ΔΨ_m_, and does not mediate any apparent toxic effects on cells at the concentrations used in the assays.

## Results

### PMI Upregulates Nrf2 and ARE-Dependent Antioxidant Genes

**Figure 1:**
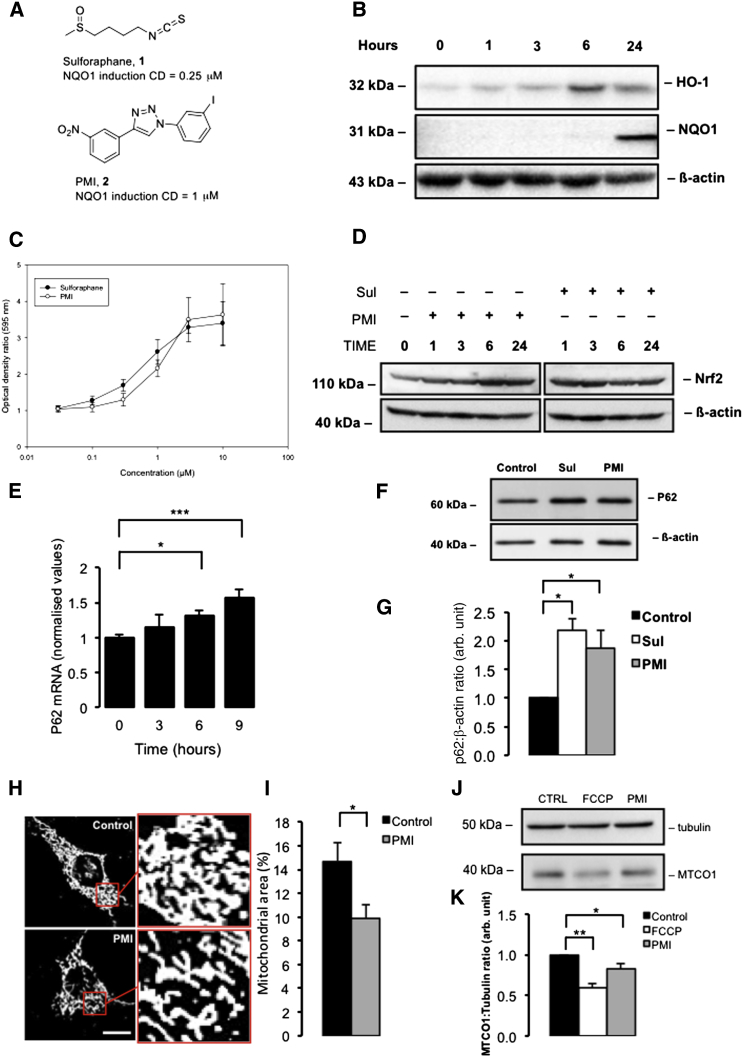
PMI Stabilizes Nrf2 and Upregulates P62 Expression Activating Mitophagy (A) Structures of compounds **1** (sulforaphane) and **2** (PMI); CD is the concentration of compound causing a doubling of the control level of NQO1 enzymatic activity. (B) Western blot to show induction of Nrf2-dependent gene products versus time in Hepa1c1c7 cells (cytoplasmic) exposed to 10 μM PMI. (C) Induction of NQO1 (NAD(P)H dependent quinone oxidoreductase-1) by compounds **1** (○) and **2** (●). (D) Western blots to demonstrate Nrf2 stabilization in cells treated with either PMI (10 μM) or, sulforaphane (1 μM) versus time (E) RT-PCR analysis for estimation of *p62* mRNA levels in MEFs following treatment with PMI versus time. Values are presented as arbitrary units normalized to 18 s RNA levels for each sample, n ≥ 3. (F) Western blot to demonstrate P62 expression in MEF cells treated with DMSO vehicle control, 10 μM PMI, or 1 μM sulforaphane for 24 hr. Β-actin is shown as a loading control. (G) Graph shows P62:β-actin ratio band density analysis, n = 3. (H) Representative confocal images of β-subunit staining to highlight mitochondrial density in MEF cells treated with DMSO vehicle control or 10 μM PMI for 24 hr. (I) Graph shows average mitochondrial area as a percentage of cell size, n ≥ 50. (J) Western blot to demonstrate reduction in MTCO1 levels following 4 hr FCCP or 24 hr PMI exposure. (K) Graph shows MTCO1:Tubulin ratio band density analysis normalized to control, n = 3. All values are mean ± SEM, ^∗^p < 0.05, ^∗∗^p < 0.01, ^∗∗∗^p < 0.001.

To investigate the effect of PMI (Figure 1A, compound 2) on expression of antioxidant proteins, levels of two Nrf2-dependent gene products—heme oxygenase-1 (HO-1) and NAD(P)H dependent quinone oxidoreductase-1 (NQO1)—were assayed in mouse Hepa1c1c7 cells over time with western blotting. In these cells, the maximal level of cytoplasmic HO-1 expression was observed after 6 hr of PMI treatment, whereas elevated cytoplasmic NQO1 levels were not detected until 24 hr after PMI exposure (Figure 1B). Additionally, the induction of NQO1 enzymatic activity after treatment with the positive control inducer sulforaphane (Figure 1A, compound 1) and PMI was assayed. Both the electrophilic Nrf2-inducing agent sulforaphane and PMI showed similar abilities to induce NQO1 activity (Figure 1A). Although the CD (concentration causing a 2-fold induction of NQO1) was greater for PMI (0.6 μM) compared to sulforaphane (0.3 μM), the maximal induction of NQO1 at 10 μM was higher for PMI (3.7-fold) than sulforaphane (3.4-fold) (Figure 1C). To further understand the kinetics of Nrf2 activation, we monitored cellular Nrf2 levels in MEFs after treatment with PMI or sulforaphane over time as an indication of Nrf2 stabilization. With PMI treatment, maximum Nrf2 levels were observed after 6 hr and remained elevated at 24 hr after exposure. In contrast, incubation with sulforaphane produced a maximum elevation of Nrf2 at 3 hr that had diminished at 6 and 24 hr, although not back to the level of the control (Figure 1D).

Encouraged by these initial findings, we sought to test the capability of PMI to upregulate the Nrf2-dependent protein P62, an autophagic adaptor, deregulation of which is linked to several neurological pathologies (Geetha et al., 2012, Salminen et al., 2012). After exposure of MEF cells to 10 μM PMI or 1 μM sulforaphane, we monitored *p62* mRNA levels over time using quantitative RT-PCR. There was a significant increase in *p62* mRNA levels after 9 hr of PMI treatment (Figure 1E). Western blot analysis of cell lysates after 24 hr revealed cytosolic P62 protein levels in PMI-treated cells had increased 1.8-fold compared to untreated cells (Figures 1F and 1G), an increase statistically indistinguishable from the effect of sulforaphane (2.1-fold). Because P62 has a major role in mitochondrial autophagy, we investigated whether pharmacologically induced P62 overexpression could influence both macro and mitochondrial type autophagy induction.

### PMI Promotes Mitochondrial LC3 Recruitment and Network Refinement

**Figure 2:**
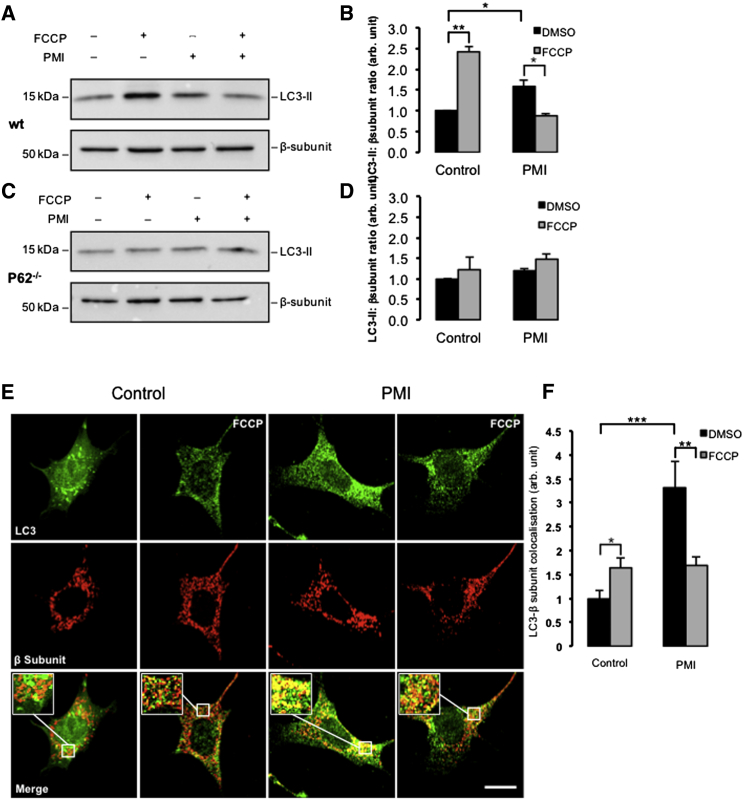
PMI Drives LC3 Mitochondrial Recruitment (A and B) Western blot highlighting increased LC3-II in the mitochondrial fraction of WT MEFs treated with FCCP (A) and PMI quantified in (B); n = 3. (C and D) Western blot highlighting no increase in LC3-II in the mitochondrial fraction of *p62*^−/−^MEFs treated with FCCP (C) and PMI quantified in (D) n = 3. (E) Representative images of LC3 localization in MEF cells treated with DMSO vehicle control or 10 μM PMI for 24 hr, before and after treatment with FCCP (20 μM) for 4 hr. Scale bar represents 10 μm. A magnification of the merge images is shown in areas demarcated by the white box. (F) Quantification of the degree of LC3:β-subunit colocalization in MEF cells. All values are mean ± SEM, ^∗^p < 0.05, ^∗∗^p < 0.01, ^∗∗∗^p < 0.001.

We monitored the conversion of LC3B-I to the lipidated LC3B-II in the cytoplasmic fraction of PMI- and vehicle-treated MEFs (Kabeya et al., 2000). Immunoblot analysis revealed no differences between the two treatments (Figures S1A and S1B available online). This experiment was repeated in the presence of Bafilomycin A1, which prevents the maturation of autophagic vacuoles as it inhibits the fusion of autophagosomes with lysosomes (Yoshimori et al., 1991), and is therefore a useful pharmacological tool to further investigate whether PMI interferes with the activation of general autophagy. However, in these experimental conditions, the level of LC3 lipidation (Klionsky et al., 2012) also remained unchanged after exposure to PMI, suggesting that PMI does not trigger any upregulation of general cellular autophagy. Although general autophagy appeared unaffected following PMI exposure, we were curious to test the effect of PMI on the specific removal of mitochondria by autophagy and hence the participation of P62 in mitophagy (Ding et al., 2010, Geisler et al., 2010, Huang et al., 2011). We initially examined the density of the mitochondrial network by immunofluorescence (Figures 1H and 1I). Cells treated with PMI for 24 hr were compared with untreated cells and a reduction in the network size measured via staining of the F_1_Fo-ATPsynthase (Abrahams et al., 1994) was noticeable in PMI-exposed cells, consistent with an alteration of mitochondrial network structure by the activation of selective autophagy. Additionally we monitored the levels of the mitochondrial inner membrane protein cytochrome c oxidase subunit I (CO1 or MTCO1) (Capaldi, 1990) after PMI and carbonyl cyanide 4-(trifluoromethoxy)phenylhydrazone (FCCP) (Heytler and Prichard, 1962) treatment as an indication of mitochondrial clearance (Figure 1J). Both PMI and FCCP treatment reduced the levels of MTCO1, although this was more pronounced with FCCP (Figure 1K). Unlike PMI treatment, exposure to sulforaphane did not cause a reduction in MTCO1 levels (Figures S1C and S1D). To further assess the role of LC3, we examined LC3-II levels in mitochondrial fractions with western blotting. Increased mitochondrial LC3-II was observed in cells treated with both PMI and FCCP (Figures 2A and 2B) whereas in *p62*^−/−^ MEF cells, this increase was not detected (Figures 2C and 2D). Additionally we monitored the colocalization of mitochondria and LC3B in MEFs using high-resolution confocal imaging (Figure 2E). Under resting conditions, cells treated with PMI showed a dramatic increase in mitochondrial LC3B colocalization (PMI-DMSO: 3.31 ± 0.54 normalized fluorescence, units [n.f.]) compared to untreated conditions (control-DMSO: 1.00 ± 0.17 n.f.). However, when mitophagy was induced with FCCP, the increase in colocalization attributed to PMI was normalized; colocalization coefficients for FCCP-stimulated cells in both controls and PMI-treated conditions were approximately equal (control-FCCP 1.64 ± 0.20; PMI-FCCP 1.69 ± 0.18 n.f.); yet, as expected, with a significant induction of mitophagy compared to untreated conditions (control-DMSO: 1.00 ± 0.17 n.f.) (Figure 2F). In cells lacking Nrf2 (*Nrf2*^*−/−*^ MEF), the observed increase in mitochondrial LC3 colocalization attributed to PMI was abolished (Figures S1E and S1F).

### PMI Acts Downstream of the PINK1/Parkin Signaling Pathway

**Figure 3:**
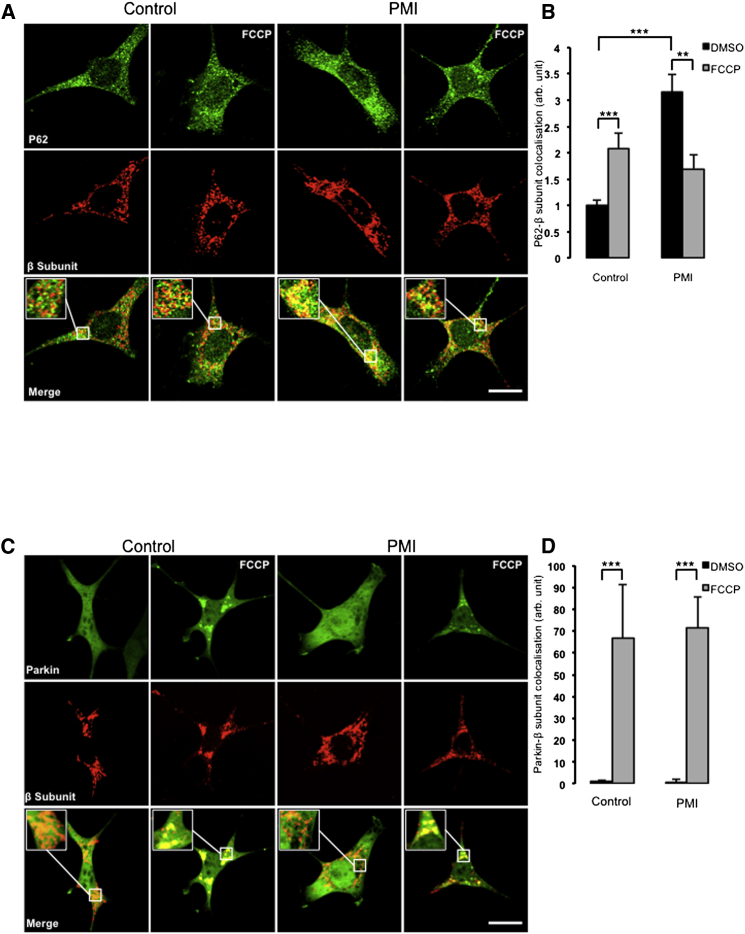
PMI-Induced Mitochondrial Recruitment of P62 Is Parkin Independent (A) Representative images of P62 localization in MEF cells treated with DMSO vehicle control or 10 μM PMI for 24 hr, before and after treatment with FCCP (20 μM) for 4 hr. Scale bar represents 10 μm. A magnification of the merge images is shown in areas demarcated by the white box. Scale bar represents 10 μm. (B) Quantification of the degree of p62:β-subunit: colocalization in MEF cells, n > 30. (C) Representative images of Parkin localization in MEF cells treated with DMSO vehicle control or 10 μM PMI for 24 hr, before and after treatment with FCCP (20 μM) for 4 hr. Scale bar represents 10 μm. A magnification of the merge images is shown in areas demarcated by the white box. Scale bar represents 1 μm. (D) Quantification of the degree of parkin:β-subunit colocalization in MEF cells, n > 30. All values are mean ± SEM, ^∗∗^p < 0.01, ^∗∗∗^p < 0.001.

LC3-containing autophagosomes can be efficiently recruited to mitochondria by P62 (Ding et al., 2010, Geisler et al., 2010, Huang et al., 2011). To investigate if PMI treatment corresponded with increased mitochondrial-associated P62, a similar confocal imaging experiment was performed (Figure 3A) to measure colocalization of P62 with the mitochondrial network. In this case, a similar trend was observed (Figure 3B): under basal conditions, PMI-treated cells showed a substantial increase in mitochondrial P62 colocalization, relative to control, whereas this effect was normalized by addition of FCCP (control-DMSO 1.00 ± 0.10; PMI-DMSO 3.15 ± 0.33; control-FCCP 2.08 ± 0.28; PMI-FCCP 1.70 ± 0.27).

**Figure 4:**
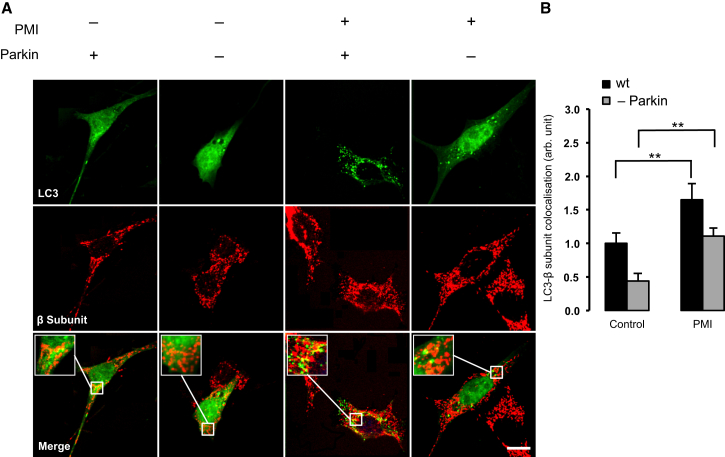
In Parkin Knockdown Cells, PMI Promotes LC3 Mitochondrial Accumulation (A) Representative images of LC3 localization in WT and Parkin knockdown MEF cells treated with DMSO vehicle control or 10 μM PMI for 24 hr. Scale bar represents 10 μm. (B) A magnification of the merged images is shown in areas demarcated by the white box. Quantification of mitochondrial LC3 localization in WT and *Parkin* knockdown MEF cells treated with DMSO vehicle control or 10 μM PMI for 24 hr, n > 30. All values are mean ± SEM, ^∗∗^p < 0.01.

**Figure 5:**
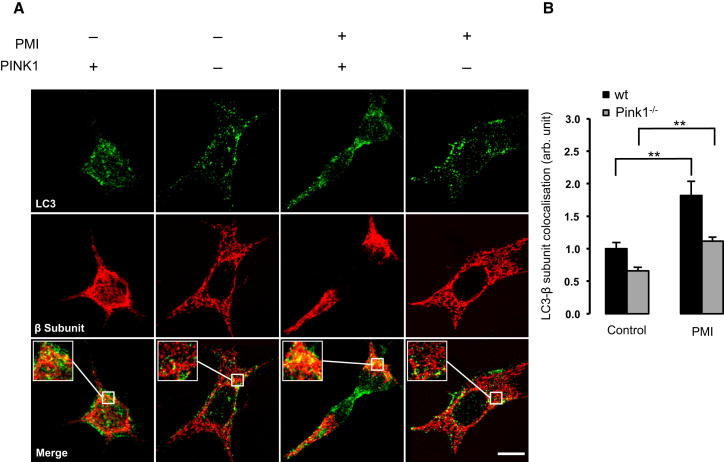
In PINK1 Knockout Cells, LC3 Is Recruited to Mitochondria by PMI (A) Representative images of LC3 localization in WT and Pink1 knockout SH-SY5Y treated with DMSO vehicle control or 10 μM PMI for 24 hr. Scale bar represents 10 μm. A magnification of the merge images is shown in areas demarcated by the white box. (B) Quantification of mitochondrial LC3 localization in WT and Pink1 knockout SH-SY5Y cells treated with DMSO vehicle control or 10 μM PMI for 24 hr, n > 30. All values are mean ± SEM, ^∗∗^p < 0.01.

During PINK1/Parkin-mediated mitophagy, mitochondrial P62 association occurs in response to Parkin mediated ubiquitination of OMM substrates (Jin and Youle, 2012). To examine whether the observed mitochondrial recruitment of P62 and LC3B were a consequence of a PMI-induced relocation of Parkin, further imaging experiments were performed (Figure 3C). In this case, MEFs incubated with PMI showed no increase in mitochondrial Parkin translocation compared to control (control-DMSO 1.00 ± 0.75; PMI-DMSO 0.88 ± 1.27). Instead, exposure to FCCP initiated a significant relocation of Parkin to mitochondria, in both untreated and PMI treated cells (control-FCCP 66.91 ± 24.22; PMI-FCCP 71.30 ± 14.38) (Figure 3D). This trend was inconsistent with that observed for LC3B and P62, suggesting that the action of PMI may occur downstream of Parkin. To further test this hypothesis, we assayed mitochondrial LC3 colocalization in Parkin knockdown MEFs and in *pink1* knockout SH-SY5Y (Figures 4 and 5). Notably, in both cases, mitophagy was increased by PMI (Figure 4, wild-type [WT]: control-DMSO 1.00 ± 0.16; PMI-DMSO 1.65 ± 0.24; Parkin K/D: control-DMSO 0.44 ± 0.12; PMI-DMSO 1.12 ± 0.13 n.f.) (Figure 5, WT: control-DMSO 1.00 ± 0.10; PMI-DMSO 1.82 ± 0.21; *pink1*^*−/−*^: control-DMSO 0.66 ± 0.05; PMI-DMSO 1.11 ± 0.06; control-FCCP 1.23 ± 0.07 PMI-FCCP 1.84 ± 0.11 n.f.).

This result suggests that PMI is still able to trigger mitophagy in the absence of PINK1 or Parkin, but also highlights that an intact PINK1/Parkin signaling pathway leads to more efficient mitochondrial clearance when triggered by PMI. This experimental outcome led us to investigate the effect of PMI on mitochondrial poly-ubiquitination.

### PMI Positively Affects Mitochondrial Poly-Ubiquitination and Coupling

**Figure 6:**
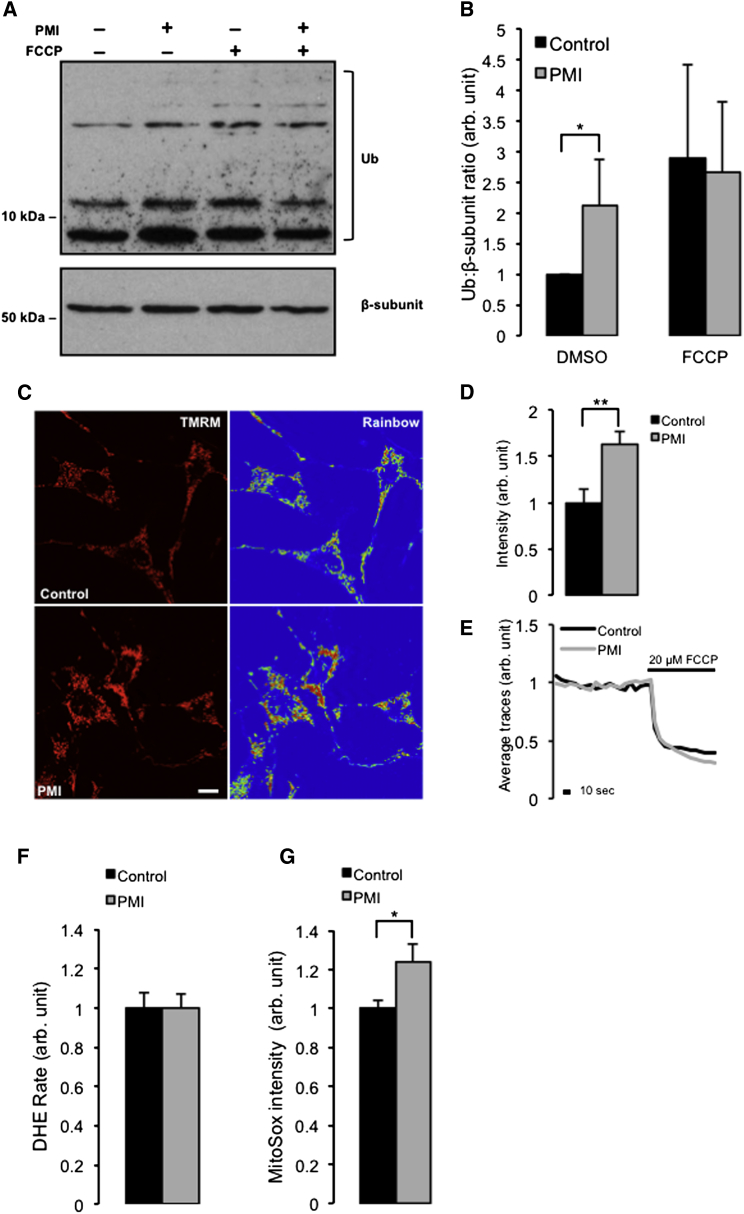
PMI Mediates Poly-Ubiquitination of Mitochondria and Increases ΔΨ_m_ (A) Western blot of mitochondrial fractions, highlighting mitochondrial ubiquitination in control or PMI-treated MEF cells before and after treatment with 20 mM FCCP. (B) The graph shows total ubiquitin band density analysis relative to β-subunit loading control, n = 3. (C) Representative confocal images depicting differences in ΔΨ_m_ when in MEF cells treated with DMSO vehicle control or 10 μM PMI for 24 hr and loaded with the cationic mitochondria selective probe TMRM (red) for 30 min. Scale bar represents 10 μm. (D and E) Mean basal TMRM fluorescence quantification n > 10 (D) and shows representative traces of the effects of FCCP on mitochondrial membrane potential in treated with DMSO vehicle control or 10 μM PMI for 24 hr (E). (F) Values of cytosolic ROS accumulation data collected in MEF cells by recording the rate of nuclear uptake of the O_2_^−^ sensitive dye, dihydroethidium (DHE), n > 30. (G) Mitochondrial ROS generation collected in MEF cells by recording fluorescence intensity of O_2_^−^ sensitive, mitochondrial-specific dye, MitoSOX, n > 20. All values are mean ± SEM, ^∗^p < 0.05 ^∗∗^p < 0.01.

Poly-ubiquitination of mitochondrial OMM proteins by ubiquitin ligases flags damaged mitochondria for destruction (Geisler et al., 2010). When the mitochondrial fractions of cells treated with PMI and FCCP were analyzed for ubiquitination (Figure 6A), an increased level was observed compared to control conditions (Figure 6B). However, as per the previous analyses, an additive effect in cells treated with both PMI and FCCP was not detected (control-DMSO 1.00 ± 0.00; PMI-DMSO 2.11 ± 0.84; control-FCCP 2.89 ± 1.51 PMI-FCCP 2.66 ± 1.09).

To further elucidate the mechanism by which PMI may be inducing mitophagy we tested aspects of mitochondrial physiology, which are directly involved in the activation of the process such as dissipation of the mitochondrial membrane potential (ΔΨ_m_). ΔΨ_m_ was monitored in PMI-treated cells with tetramethylrhodamine, methyl ester (TMRM), using signal intensity as a function of membrane potential (Figures 6C and 6D). Notably, cells incubated with PMI had a higher resting ΔΨ_m_ (2.22 ± 0.14 n.f.) when compared to control cells (3.62 ± 0.31 n.f.), whereas the rate of FCCP-induced depolarization of ΔΨ_m_ was not affected (Figure 6E).

Along with the measurements of ΔΨ_m_, we then explored redox signaling. An excess of reactive oxygen species (ROS) may function as an autophagy trigger (Scherz-Shouval and Elazar, 2011) and dysfunctional mitochondria that overproduce ROS, are indeed selectively targeted by mitophagy (Kim et al., 2007). We therefore investigated the effect of PMI on mitochondrial and cellular ROS. Superoxide production was assayed in cells treated with PMI for 24 hr by monitoring the oxidation of the fluorescent probes mitoSOX and DHE to monitor mitochondrial and cytosolic ROS, respectively. While no disparity in cytosolic ROS production (Figure 6F) between treated and control cells was observed, ROS within mitochondria (Figure 6G) appeared moderately increased in cells exposed to PMI (control: 1.00 ± 0.04; PMI: 1.24 ± 0.09). A possible explanation of this is that PMI, by promoting segregation of defective mitochondria from the network via autophagy, could leave only those that are fully polarized, resulting in an overall increased rate of respiration within the remaining mitochondria. This may lead to the observed increase in mitochondrial ROS. This excess however is undetectable at the cytosolic level, possibly due to the concomitant upregulation of antioxidant mechanisms.

## Discussion

The research devoted to the mechanisms underlying mitophagy has not been matched by the development of pharmacological tools to regulate the process. The prototypical approach to trigger mitophagy is based on the application of chemical ionophores: FCCP or carbonyl cyanide m-chlorophenyl hydrazone. These agents permeabilize the inner mitochondrial membrane to H^+^, destroying the H^+^ gradient, and in doing so, the electron transport chain of the oxidative phosphorylation system (Hirose et al., 1974). Although this protocol triggers fast and efficient depolarization of the organelle and PINK1/Parkin pathway activation (Kawajiri et al., 2010, Matsuda et al., 2010, Narendra et al., 2010), it has little physiological significance and has a number of adverse downstream effects (Padman et al., 2013). Recently, Jin and Youle have elegantly shown that the accumulation of misfolded proteins in mitochondria may suffice as a physiological molecular trigger for mitophagy activation without collapsing the ΔΨ_m_ (Jin and Youle, 2013). Additionally, the discovery that cardiolipin externalization on mitochondria acts as a surface marker of dysfunctional organelles (Chu et al., 2013) highlights the need for pharmacological tools acting by a different mechanism to ionophores, to better dissect the contribution of inner mitochondrial pathways to the efficiency of mitophagy.

We therefore identified a compound that promotes the upregulation of P62 via stabilization of Nrf2 within treated cells (Baird and Dinkova-Kostova, 2011). This prototype molecule, which we called PMI, increases the expression of Nrf2-dependent gene products including P62, HO1, and NQO1 without perturbing mitochondrial function, the latter effect appears to distinguish the compound from some of the reported effects of sulforophane that exploits the same pathway. PMI increases the recruitment of LC3 to mitochondria without alteration to its lipidation or flux, an indication that this is not an effect on macro-autophagy (Figures 2B, 2F, 3B, and S1B). The absence of increased LC3 in the mitochondrial fractions of PMI-treated *p62*^*−/−*^ MEFs serves to confine the effects to those associated with the presence of P62 (Figures 2B and 2D). This is further supported by the data from *Nrf2*^*−/−*^ MEFs in which PMI-induced recruitment of LC3 to mitochondria is lost as expected, possibly due to the diminished P62 levels relative to the WT cells (Figures S1E and S1F).

Notably, the mitochondrial relocalization of P62 and LC3 triggered by PMI was partially reduced when FCCP was added, suggesting that these two compounds may antagonize each other. By increasing P62 levels, PMI may promote the maximal recruitment of the “available” P62 to mitochondria, and, although the integrity of the PINK1/Parkin pathway (Kawajiri et al., 2010) is important for its action (see data in Figures 4 and 5), this is not its first site of intervention (Figures 3C and 3D). In cells challenged by PMI, FCCP may therefore drive a further stress by depolarizing ΔΨ_m_ that may become unsustainable for the autophagic machinery, thus diminishing the efficacy of PMI (Figures S1A and S1B). Nevertheless, simultaneous exposure to FCCP and PMI does not affect mitochondrial ubiquitination (Figures 6A and 6B), which was increased by PMI in line with the evidence indicating that P62 promotes a more dynamic ubiquitination of dysfunctional mitochondria by activating the ubiquitin ligase activity of TRAF6 that binds and stabilizes PINK1 on depolarized mitochondria (Murata et al., 2013, Wooten et al., 2005).

Although we cannot rule out the possibility that the abrupt collapse of mitochondrial respiration caused by FCCP could be detrimental to PMI activity, the data obtained in (1) MEFs transiently downregulated for Parkin (MEF Parkin KD) as well as in (2) SH-SY5Y cells devoid of PINK1 (SH-SY5Y *pink1* KO) highlight an alternative mechanism of action for PMI that is still capable of promoting mitochondrial recruitment of LC3 even if the PINK1/Parkin pathway is defective (Figures 4 and 5).

**Figure 7:**
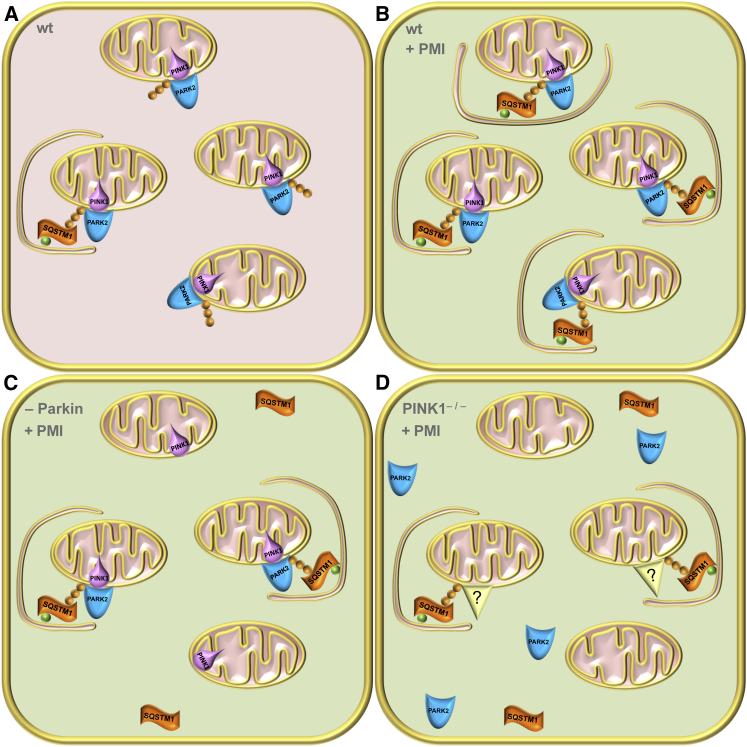
The Proposed Working Model for PMI (A) In healthy WT cells, a proportion of mitochondria will be destined for destruction due to age, damage, or dysfunction. Parkin-mediated ubiquitination (orange spheres) primes these mitochondria to enter the autophagic pathway and available P62 links them to LC3 (green spheres) and the growing autophagosome. (B) In PMI-treated cells, P62 is more abundant therefore able to drive mitochondria into autophagy with increased efficiency. (C) In cells where Parkin expression is reduced, a reduced number of mitochondria are primed for autophagy, so although P62 is overexpressed, the efficiency of mitophagy is reduced. (D) Finally, in cells devoid of PINK1, Parkin is not recruited to mitochondria; however, redundant/alternative ubiquitin ligases may still be capable of ubiquitinating mitochondria, which can then be driven into autophagy by P62, albeit with reduced efficiency.

In the working model depicted in Figure 7, we suggest that PMI determination of mitophagy may be made possible by redundant mitochondrial ubiquitination mechanisms that we have not identified but that could be the similar to those exploited by cells endogenously devoid of Parkin (e.g., HeLa) but that still preserve mitochondrial quality control (Denison et al., 2003, Pawlyk et al., 2003). The upregulation of the P62 pathway may represent a compensatory mechanism for defective mitophagy, making compounds related to PMI potential tools to restore mitophagy in pathological conditions in which the PINK1/Parkin pathway is impaired such as in Parkinson disease (Chu, 2010, Narendra et al., 2009). The null effect on mitophagy recorded for sulforaphane, despite its upregulation of P62 is intriguing. Sulforaphane and PMI have different time-dependent effects on Nrf2 stabilization, PMI has a slower onset of action, but a more sustained effect (Figure 1D), which may contribute to their differing effects on mitophagy. There is some evidence from the literature that sulforaphane has additional effects on mitochondria and mitochondrial biogenesis (Negrette-Guzmán M. et al., 2013), so it is possible that these may influence the mitophagic process. Alternatively, events in addition to P62 overexpression may be responsible for the effects of PMI. Further experiments will be required to fully elucidate and clarify the mode of action, and the factors that distinguish PMI from sulforaphane in the area of mitophagy.

PMI-treated mitochondria display an increased superoxide metabolism, (Suski et al., 2012) (Figure 6G) which, in light of the parallel effects on mitochondrial network appearance (Figure 1H) and ΔΨ_m_ (Figures 6C and 6D), could indicate an improved oxidative-respiratory capacity; data that are consistent with recent reports (Holmström et al., 2013). The accompanying respiratory metabolic by-products remain undetectable in the cytoplasm, possibly due to the Nrf2-mediated upregulation of antioxidant responses as demonstrated by increases in ARE-dependent HO1 and NQO1 expression (Figures 1B–1D).

Based on these in vitro findings, PMI is a molecule that drives mitochondria to a process of quality control without compromising the bio-energetic competence of the whole network but exposing just those organelles to be recycled. By acting alternately from FCCP, PMI could be a useful tool to define the contribution of molecules regulating mitophagy and illuminate the coordination between mitochondrial and nonmitochondrial events that drive execution of the process.

## Significance

**Mitochondrial quality control is a fundamental process in cellular homeostasis, and its deficiency is linked to several neurodegenerative diseases and cancers. Despite this, the discrete mechanisms, regulatory pathways, and impact on cellular physiology are still far from being elucidated. The current canonical methods to trigger mitophagy in vitro typically involve the abrupt depolarization of mitochondrial membrane potential (ΔΨ**_**m**_ **) using ionophores such as carbonyl cyanide m-chlorophenyl hydrazone or FCCP that mediate various detrimental effects on several other cellular processes. PMI is a pharmacological agent that can trigger mitophagy while leaving ΔΨ**_**m**_ **intact. Therefore, PMI has enormous potential as a tool to further study the finely tuned process of mitochondrial quality control and dysregulation in disease states in a more physiologically relevant background.**

## Experimental Procedures

### Chemistry and Analytical Data

Sulforaphane was purchased from Sigma-Aldrich (S6317). PMI (1-(3-iodophenyl)-4-(3-nitrophenyl)-1,2,3-triazole) was synthesized from commercially available starting materials in four solution-phase synthetic steps.

^1^H and ^13^C nuclear magnetic resonance (NMR) spectra were recorded on a Bruker Avance 400 instrument using solvent residuals as internal references. The following abbreviations are used: singlet (s), doublet (d), doublet of doublets (dd), triplet (t), doublet of triplets (dt), and multiplet (m). UCL School of Pharmacy provided high-resolution mass spectrometry and elemental analysis services. Melting points (mp, uncorrected) were determined using a Stuart Melting Point Apparatus SMP30 (Bibby Scientific). Thin-layer chromatography analysis was carried out on silica gel (Merck 60F-254) aluminum backed plates with depiction at 254 and 365 nm. Preparative flash chromatography was carried out with Merck silica gel (Si 60, 40–63 μm). Reagents, chemicals, and dry solvents (DMF) were purchased from Sigma-Aldrich unless otherwise stated. Liquid chromatography-mass spectrometry (LC-MS) analyses were performed on a Waters Micromass ZQ instrument coupled to a Waters 2695 high-performance liquid chromatography (HPLC) with a Waters 2996 PDA (LC-MS instrument). Waters Micromass ZQ parameters used were capillary (kV), 3.50; cone (V), 30; extractor (V), 3.0; source temperature (°C), 120; desolvation temperature (°C), 350; cone flow rate (l/hr), 80; desolvation flow rate (l/hr), 650. Analytical reverse-phase HPLC was carried out on a Phenomenex Onyx Monolithic C-18 column 50 × 4.6 mm. HPLC experiments were performed with gradient conditions: 95% solvent A, 5% solvent B for 1 min, then from 5% B to 95% B over 4 min (total run time 5 min, flow rate 1.5 ml/min) (solvent A [H_2_O containing 0.1% v/v formic acid] and solvent B [MeCN containing 0.1% v/v formic acid]).

#### 1-Ethynyl-3-Nitrobenzene

To a solution of 1-bromo-3-nitrobenzene (2.0 g, 9.90 mmol, 1.0 equiv.) in dry DMF (5 ml) was added to diethylamine (10 ml); nitrogen gas was bubbled through the resulting solution for 20 min. CuI (8.4 mg, 0.04 mmol, 0.4%mol), Pd(PPh_3_)_4_ (125.9 mg, 0.11 mmol, 1.1%mol), and trimethylsilylacetylene (1.53 ml, 11.00 mmol, 1.11 equiv.) were then added and the resulting mixture was submitted to the following microwave heating sequence: prestirring for 30 s, then 1 hr at 120°C (Wang et al., 2007). The resulting mixture was poured into aqueous HCl (1 M, 60 ml) and extracted with DCM. The organic was washed with aqueous HCl (1 M, 50 ml) and brine (saturated solution, 50 ml), dried over MgSO_4_, filtered, and concentrated under reduced pressure. The residue was purified by column chromatography on silica gel (hexane/ethyl acetate 9/1) to afford 3-(trimethylsilylethynyl)nitrobenzene as a yellow solid (1.05 g, 48.5% chemical yield). R_f_(hexane/EtOAc 9:1) = 0.55; ^1^H-NMR (400 MHz, CDCl_3_): δ (ppm) 8.3 (t, *J* = 1.6 Hz, 1H), 8.16 (ddd, *J* = 1.2, 2.2, 8.0 Hz, 1H), 7.75 (dt, *J* = 1.2, 8.0 Hz, 1H), 7.48 (t, *J* = 8.0 Hz, 1H), 0.27 (s, 9H); ^13^C-NMR (100 MHz, CDCl_3_): δ (ppm) 148.0, 137.5, 129.2, 126.8, 125.0, 123.1, 102.1, 97.6, 0 (3C); and LC-MS 100%, 3.60 min.

3-(trimethylsilylethynyl)nitrobenzene (317.0 mg, 1.45 mmol, 1.0 equiv.) was dissolved in methanol (15 ml). K_2_CO_3_ (359.0 mg, 2.6 mmol, 1.8 equiv.) was added and the suspension was stirred under N_2_ at RT for 2.5 hr. The mixture was filtered, the filtrate evaporated, and the residue taken up in water and extracted with EtOAc. The organic phase was washed with brine, dried over MgSO_4_, filtered, and concentrated under reduced pressure to afford1-ethynyl-3-nitrobenzene as a brown oil (180.0 mg, 85% chemical yield). R_f_ (hexane/EtOAc 9:1) = 0.47; ^1^H-NMR (400 MHz, CDCl_3_): δ (ppm) 8.32 (t, *J* = 2.0 Hz, 1H), 8.19 (ddd, *J* = 1.2, 2.4, 8.0 Hz, 1H), 7.78 (dt, *J* = 1.2, 8.0 Hz, 1H), 7.51 (t, *J* = 8.0 Hz, 1H), 3.22 (s, 1H); ^13^C-NMR (100 MHz, CDCl_3_): δ (ppm) 148.1, 137.7, 129.4, 127.0, 123.9, 123.5, 81.1, 79.9; and LC-MS 100%, 3.15 min.

#### 1-Azido-3-Iodobenzene

3-Iodo-aniline (500.0 mg, 0.275 ml, 2.28 mmol, 1.0 equiv.) was suspended in water (10 ml) and aqueous HCl (1 ml, 10% v/v) was added and the solution cooled to 0°C. A solution of NaNO_2_ (189.0 mg, 2.74 mmol, 1.2 equiv.) in water (2 ml) was added dropwise at 0°C and the mixture stirred for a further 20 min (Faucher et al., 2002). A solution of NaN_3_ (223.0 mg, 3.43 mmol, 1.5 equiv.) in water (2 ml) was added dropwise at 0°C and the resulting suspension stirred for a further 3 hr. The solution was extracted with Et_2_O; the organic phase washed with water and saturated brine, then dried over MgSO_4_, filtered, and evaporated to afford 1-azido-3-iodobenzene as an orange oil (525.8 mg, 94% chemical yield). ^1^H-NMR (400 MHz, CDCl_3_): δ (ppm) 7.46 (dt, *J* = 1.2, 7.6 Hz, 1H), 7.37 (t, *J* = 2.0 Hz, 1H), 7.06 (t, *J* = 8.0 Hz, 1H), 6.98 (ddd, *J* = 1.2, 2.0, 8.0 Hz, 1H); ^13^C-NMR (100 MHz, CDCl_3_): δ (ppm) 141.3, 133.9, 131.0, 127.9, 118.4, 94.6.

#### 1-(3-Iodophenyl)-4-(3-Nitrophenyl)-1,2,3-Triazole

1-Ethynyl-3-nitrobenzene (40.0 mg, 0.27 mmol, 1.0 equiv.) was suspended in *tert*-butanol (1.1 ml). An aqueous solution of CuSO_4_ (2.2 mg, 9.0 μmol, 0.033 equiv.; i.e. 0.54 ml from a 4.12 mg/ml solution in water), an aqueous solution of ascorbic acid (4.8 mg, 0.027 mmol, 0.1 equiv.; i.e. 0.55 ml from an 8.7 mg/ml solution in water) and 1-azido-3-iodobenzene (66.6 mg, 0.27 mmol, 1.0 equiv.) were then added. The mixture was submitted to the following microwave heating sequence: pre-stirring for 30 s, followed by stirring at 130°C for 30 min. The resulting suspension was diluted with water and filtered; the residue was washed with water and dried under vacuum to afford 1-(3-iodophenyl)-4-(3-nitrophenyl)-1,2,3-triazole as a yellow solid (57.0 mg, 53% chemical yield). mp 211-216°C (decomp.); ^1^H-NMR (400 MHz, DMSO-d_6_): δ (ppm) 9.65 (s, 1H), 8.74 (t, *J* = 2.0 Hz, 1H), 8.39 (dt, *J* = 1.2, 8.0 Hz, 1H), 8.35 (t, *J* = 2.0 Hz, 1H), 8.25 (ddd, *J* = 0.8, 2.0, 8.0 Hz, 1H), 8.03 (ddd, *J* = 0.8, 2.0, 8.0 Hz, 1H), 7.90 (dt, *J* = 1.2, 8.0 Hz, 1H), 7.83 (t, *J* = 8.0 Hz, 1H), 7.44 (t, *J* = 8.0 Hz, 1H);^13^C-NMR (125 MHz, DMSO-d_6_): δ (ppm) 148.3, 145.3, 137.4, 137.2, 131.75, 131.7, 131.2, 130.7, 128.0, 122.8, 121.1, 119.5, 119.2, 95.4; LC-MS 100%, 3.68 min; HRMS (TOF ES+): calculated for C_14_H_10_IN_4_O_2_: 392.9849, found: 392.9846; elemental analysis: calculated for: C_14_H_9_IN_4_O_2_: C 42.88%, H 2.31%, N 14.29%, found: C 42.86%, H 2.30%, N 14.01%.

### Cell Culture and Transfections

MEFs and SH-SY5Y (SHY) cells were maintained at 37°C under humidified conditions and 5% CO_2_ and grown in Dulbecco’s modified Eagle medium (Life Technologies, 41966-052) supplemented with 10% fetal bovine serum (Life Technologies, 10082-147), 100 U/ml penicillin, and 100 mg/ml streptomycin (Life Technologies, 15140-122). Cells plated on glass coverslips/10 cm dishes at a 10%–20% confluence 1 day prior to transfection were transiently transfected with the genes of interest or siRNA using a standard Ca^2+^ phosphate method as described previously (Morelli et al., 2003). Cells were used in experiments 36–48 hr after transfection. To knock down *parkin* expression, species-dependent pre-designed siRNA was used—Murine *Park2* target sequence: ACCATTGGGCCTGCTGGTCTA (QIAGEN, SI01369599). Experiments, unless otherwise indicated, were performed in Dulbecco’s modified Eagle medium supplemented with 10% fetal bovine serum or in the following recording medium (RM): 125 mM NaCl, 5 mM KCl, 1 mM NaH_2_PO_4_, 20 mM HEPES, 5.5 mM glucose, 5 mM NaHCO_3_, and 1 mM CaCl_2_, pH 7.4. The murine hepatoma Hepa1c1c7 cell line was obtained from the European Collection of Cell Culture. The cells were grown as monolayers and maintained by regular passage in α-MEM (Hepa1c1c7 cell line) supplemented with 2 mM L-glutamine, 100 units/ml penicillin and 100 μg/ml streptomycin and 10% heat-inactivated fetal bovine serum, cultured at 37°C in a water vapor saturated atmosphere with 5% CO_2_.

### NQO1 Activity Assay

The method used is a modification of the procedure described by Fahey et al. (2004). Hepa1c1c7 cells were seeded in 96-well plates with a cell density of 2 × 10^4^ cells/200 μl per well. After 12 hr, the cells were treated with compound or vehicle (final DMSO concentration 0.1% DMSO) and incubated for 24 hr. The culture medium was aspirated and cells lysed using 50 μl/well lysis buffer (0.1% Tween20 in 2 mM EDTA [pH 7.5]) and the plate shaken at room temperature for 15 min. The enzyme reaction mixture (200 μl; 25 mM Tris buffer [pH 7.5] containing BSA [0.067%], Tween20 (0.01%), FAD (5 μM), G6P (1 mM), NADP (30 μM), G6P dehydrogenase (40 units), MTT (0.03%), and menadione (50 μM)) was added to each well. After 5 min at room temperature, 40 μl of the stop solution (10% SDS, final concentration 1.5% SDS) was added to each well and the plate shaken for 5 s. The absorbance at 595 nm was measured. The background optical density was measured using wells containing tissue culture medium, lysis buffer, and enzyme and stop solutions without Hepa1c1c7 cells. The optical density values at 595 nm were averaged and the background corrected ratio of optical densities (compound treated/control) was calculated.

### ΔΨ_m_ Measurements

Cells were loaded with 100 nM tetramethyl rhodamine methyl ester (TMRM) (Sigma-Aldrich, T5428) in recording medium (125 mM NaCl, 5 mM KCl, 1 mM NaH_2_PO_4_, 20 mM HEPES, 5.5 mM glucose, 5 mM NaHCO_3_, and 1 mM CaCl_2_, pH 7.4) for 30 min at 37°C. The TMRM dye accumulates in mitochondria, and its signal intensity is a function of potential (Scaduto and Grotyohann, 1999). Cells were washed once in recording medium then transferred to the Zeiss LSM 510 confocal microscope (40× objective) for imaging. After several minutes of continuous recording at basal conditions, 1 μM FCCP was added to induce depolarization. Settings were kept constant between experiments. Mitochondrial regions of interest were selected and the corresponding TMRM fluorescence intensities calculated.

### Reactive Oxygen Species Analysis

Cells were incubated with 5 μM dihydroethidium (Life Technologies, D-1168) or 5 μM mitoSOX (Life Technologies, M-36008) in recording medium (125 mM NaCl, 5 mM KCl, 1 mM NaH_2_PO_4_, 20 mM HEPES, 5.5 mM glucose, 5 mM NaHCO_3_, and 1 mM CaCl_2_, pH 7.4) for 30 min at 37°C. Cells were washed once in recording medium then transferred to a Zeiss LSM 510 confocal microscope (40× objective) for imaging and fluorescence intensity was measured through continuous recording for at least 10 min. Settings were kept constant between experiments. Mitochondrial ROIs were selected and the corresponding fluorescence intensities were calculated.

### Subcellular Fractionation

Cells were lysed in cold isotonic buffer (250 mM sucrose, 10 mM KCl, 1.5 mM MgCl_2_, 1 mM EDTA, 1 mM EGTA, 20 mM HEPES, pH 7.4) containing protease inhibitor cocktail (Roche, 05892791001) by passing through a 26 gauge needle ten times using a 1 ml syringe followed by 20 min incubation on ice. Unbroken cells and nuclei were removed by centrifugation at 800 × *g* for 5 min at 4°C. Supernatants were transferred to fresh tubes and centrifuged at 10,000 × *g* for 10 min at 4°C, Subsequent supernatants were collected as the cytosolic fractions while mitochondrial pellets were washed once in cold isotonic buffer then centrifuged at 10,000 × *g* for 10 min at 4°C. Finally, mitochondrial pellets were lysed in lysis buffer (150 mM NaCl, 1% v/v Triton X-100, 20 mM Tris pH 7.4) for 30 min on ice.

### Western Blotting

Sample proteins were quantified using a BCA protein assay kit (Fisher Scientific, 13276818). Equal amounts of protein (10–30 μg for whole cell lysates/cytosolic fractions; 10 μg for mitochondrial fractions) were resolved on 10% or 12% SDS-PAGE gels and transferred to nitrocellulose membranes (Fisher Scientific, 10339574). The membranes were blocked in 3% nonfat dry milk in 50 mM Tris, 150 mM NaCl, 0.05% Tween 20, pH 7.5 (TBST) for 1 hr, then incubated with the appropriate diluted primary antibody at 4°C overnight: rabbit α-LC3 (Abcam, ab48394) 1:1,000; mouse α-P62/SQSTM1 (Abcam, ab56416) 1:20,000; α-GAPDH-Hrp conj. 1:50,000 (Abcam, ab9484); α-ubiquitin (Abcam, ab7780) 1:10,000, mouse α-parkin (Abcam, ab77924) 1:1,000. Membranes were washed in TBST (3 × 15 min at room temperature [RT]) and then incubated with corresponding peroxidase-conjugated secondary antibodies (Dako, P0447, P0448) for 1 hr at RT. After further washing in TBST, blots were developed using an ECL Plus western blotting detection kit (Fisher Scientific, 12316992). Immunoreactive bands were analyzed by performing densitometry with ImageJ software.

Blots from Hepa1c1c7 cells were obtained as follows. Following treatment with 10 μM PMI, cells were harvested at different time intervals. Nuclear and cytosolic fractions were obtained using NE-PER Nuclear and Cytoplasmic Extraction Reagents (Thermo Scientific, 78833). Proteins were separated by electrophoresis on 10% SDS-PAGE gels and transferred onto nitrocellulose membranes. The blots were blocked with 1% skimmed milk and probed overnight with the primary antibodies: Heme Oxygenase 1 (Santa Cruz Biotechnology, sc-10789) polyclonal antibody, NQO1 (Santa Cruz Biotechnology, sc-271116) monoclonal antibody, or β-actin (Santa Cruz Biotechnology, sc-130657) polyclonal antibody. Following a 2 hr incubation period with peroxidase-conjugated secondary antibodies, proteins were detected using enhanced chemiluminescence (Fisher Scientific, 12316992).

### Immunofluorescence

Cells were fixed in 4% PFA (10 min, RT) followed by three 5-minute washes in PBS. Permeabilization was performed with 0.5% Triton-X in PBS (10 min, RT) followed by washing. Blocking was carried out for 1 hr at RT in 10% goat serum and 3% BSA in PBS. Primary antibody incubations were conducted overnight for 16 hr at 4°C in blocking solution as described. After a further wash step, secondary antibodies were incubated for 1 hr in blocking solution, before a final wash step. Cells were then mounted on slides with DAPI mounting medium (Abcam, ab 104139). Cells were stained with the following primary antibodies: mouse α-β-subunit (Abcam, ab14730) 1:1,000; α-P62/SQSTM1 (Abcam, ab56416) 1:500; α-LC3 (Abcam, ab48394) 1:50, and the following secondary antibodies: α-mouse Alexa 555 (Life Technologies, A21424) 1:1,000; α-rabbit Alexa 488 (Life Technologies, A11008) 1:1,000.

### Quantitative Real-Time PCR

Total RNA was extracted from the cultured cells using Tri-Reagent (Sigma) and purified using an RNeasy Mini Kit (QIAGEN). Spectrophotometry was used to quantify and determine purity (260/280nm ratio) of the total RNA (Nanodrop, LabTechnologies). One microgram of total RNA was used to synthesize cDNA in a 20 μl reaction volume as described using the QuantiTect Reverse Transcription kit (QIAGEN). Gene transcripts were amplified by RT-PCR using commercially available primer against mouse *p62/sqstm1* (Sigma KiCqStart SYBR Green Primer pair:: M_Sqstm1_3).

Quantitative real-time PCR reactions were performed in the Chromo 4 light cycler (Biorad). The absolute quantification method was used whereby the level of gene expression was expressed as a copy number. A standard curve was generated using known amounts of the DNA PCR product of the gene. A volume of 2 μl of the cDNA product from the reverse transcription reaction was used in a total volume of 20 μl SYBR green detection (QIAGEN). The SYBR green PCR reaction mix constituted of a final concentration of 2.5 mM MgCl_2_, 0.1 μM of each primer with the reaction consisting of the following: an initial 15 min denaturation step at 95°C followed by denaturation at 94°C for 15 s, annealing at 55°C for 25 s, and extension at 72°C for 15 s repeated for 40 cycles. The DNA standards and samples were run in the same 96-well PCR plate and the average of each duplicate value was used for subsequent statistical analyses. All PCR products were checked for specificity and purity from a melting curve profile performed by the lightcycler software at the end of each run.

### Statistical Analysis

Data are presented as mean ± SEM. One-way ANOVA was used in multiple group comparisons with Bonferroni’s post hoc test to compare two data sets within the group and a p value less than 0.05 was considered significant. All analyses were performed in Microsoft Office Excel 2007. ^∗^p < 0.05, ^∗∗^p < 0.01, ^∗∗∗^p < 0.001.

## Supplemental Information

Document S1. Figure S1

Document S2. Article plus Supplemental Information
