# Supplementary material for: PMI: A ΔΨm Independent Pharmacological Regulator of Mitophagy
Source: Chem Biol. 2014 Nov 20;21(11):1585–96. doi: 10.1016/j.chembiol.2014.09.019 (PMC4245710; doi:10.1016/j.chembiol.2014.09.019)
Supplement: Document S1. Figure S1 [file mmc1.pdf]

Chemistry & Biology, Volume *21*

Supplemental Information

PMI: A $\Delta\Psi_m$ Independent Pharmacological Regulator of Mitophagy

Daniel A. East, Francesca Fagiani, James Crosby, Nikolaos D. Georgakopoulos, Hélène Bertrand, Marjolein Schaap, Adrian Fowkes, Geoff Wells, and Michelangelo Campanella

Figure S1

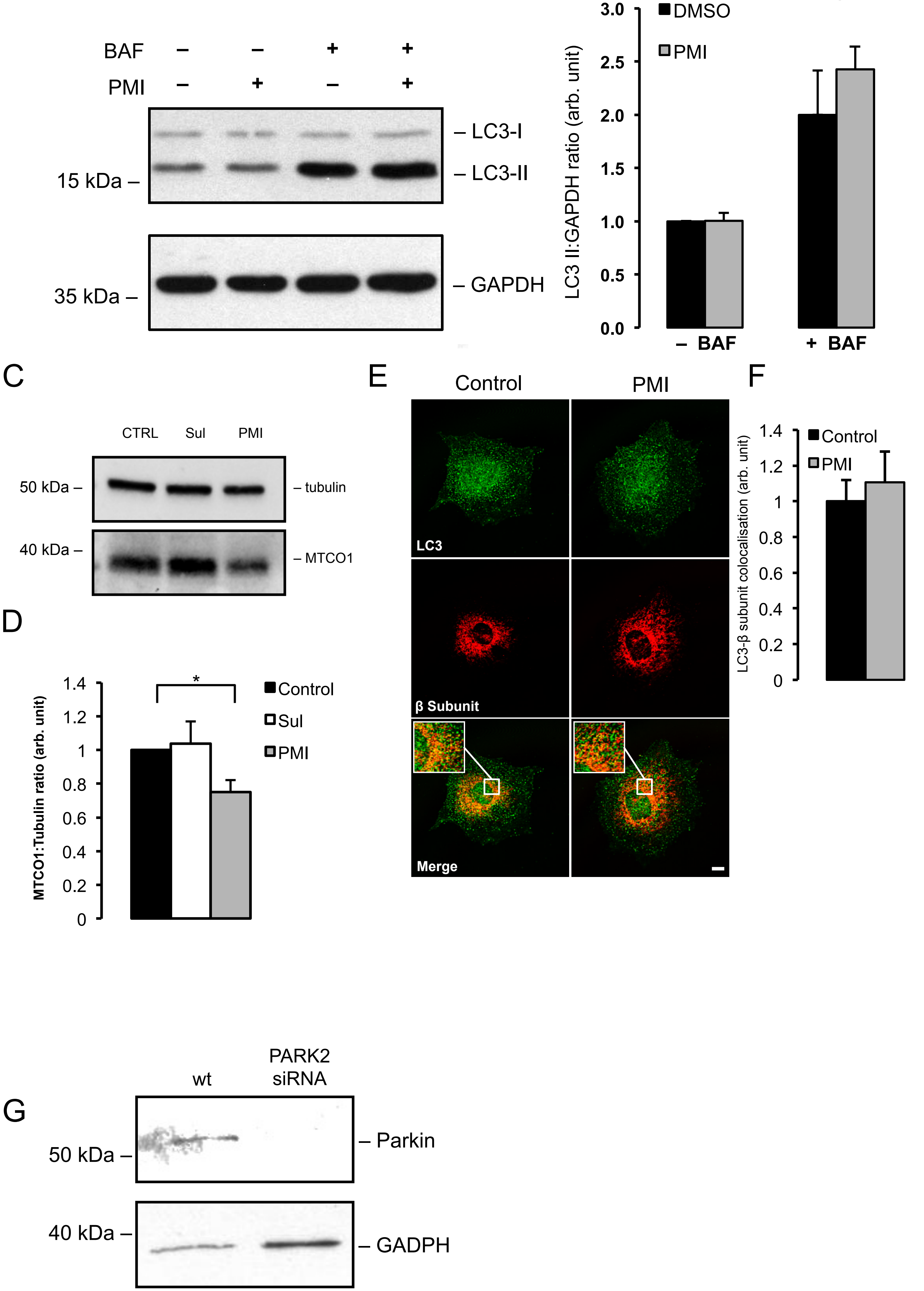
A
BAF – – + +
PMI – + – +
– LC3-I
– LC3-II
15 kDa –
35 kDa –
– GAPDH
B
DMSO
PMI
LC3 II:GAPDH ratio (arb. unit)
3.0
2.5
2.0
1.5
1.0
0.5
0.0
– BAF
+ BAF
C
CTRL Sul PMI
50 kDa –
– tubulin
40 kDa –
– MTCO1
D
MTCO1:Tubulin ratio (arb. unit)
1.4
1.2
1
0.8
0.6
0.4
0.2
0
*
Control
Sul
PMI
E
Control
PMI
LC3
β Subunit
Merge
F
LC3-β subunit colocalisation (arb. unit)
1.4
1.2
1
0.8
0.6
0.4
0.2
0
Control
PMI
G
wt
PARK2 siRNA
50 kDa –
– Parkin
40 kDa –
– GADPH

Figure S1

(A) Western blot analysis of LC3-I and LC3-II expression levels in MEF cells treated with DMSO vehicle control or 10 μM PMI in the presence of absence of bafilomycin A1 (100 nM) for 24 hours and quantified in (B) The data indicate that that PMI does not increase the activation of general autophagy as measured by the conversion of LC3-I to LC3-II, n=3. (C) Western blot to compare MTCO1 levels following 24 hours PMI (10 μM) or sulforaphane (1 μM) exposure and is quantified in (D) n=3. The data demonstrates that PMI causes a decrease in levels of mitochondrial protein MTCO1, indicative of mitophagy, whereas sulforaphane does not. (E) Representative images of LC3 localisation in $Nrf2^{-/-}$ MEF cells treated with DMSO vehicle control or 10 μM PMI for 24 hours. Scale bar= 10 μm. A magnification of the merge images is shown in areas demarcated by the white box. Mitochondrial LC3 colocalisation is quantified in (F) n=>10. The data shows that in the absence of Nrf2, the affect of PMI on mitochondrial LC3 localisation is abolished. (G) Western Blot demonstrating Parkin knock-down in cell lysates from MEFs transiently transfected with PARK2 siRNA. All values are mean ± SEM, *p<0.05
